# Association between Social Integration and Face Mask Use Behavior during the SARS-CoV-2 Pandemic in Japan: Results from U-CORONA Study

**DOI:** 10.3390/ijerph18094717

**Published:** 2021-04-28

**Authors:** Nobutoshi Nawa, Yui Yamaoka, Yuna Koyama, Hisaaki Nishimura, Shiro Sonoda, Jin Kuramochi, Yasunari Miyazaki, Takeo Fujiwara

**Affiliations:** 1 Department of Medical Education Research and Development, Tokyo Medical and Dental University, Tokyo 113-8519, Japan; 2 Department of Global Health Promotion, Tokyo Medical and Dental University, Tokyo 113-8519, Japan; yui.yamaoka@gmail.com (Y.Y.); 140362ms@tmd.ac.jp (Y.K.); hisaaki.nishimura@gmail.com (H.N.); fujiwara.hlth@tmd.ac.jp (T.F.); 3 Kuramochi Clinic Interpark, Utsunomiya, Tochigi 321-0114, Japan; sonopulm@gmail.com (S.S.); raouyuria@gmail.com (J.K.); 4 Department of Respiratory Medicine, Tokyo Medical and Dental University, Tokyo 113-8519, Japan; miyazaki.pilm@tmd.ac.jp

**Keywords:** face mask use, communicable diseases, social integration, coronavirus infections

## Abstract

Face mask use is a critical behavior to prevent the spread of SARS-CoV-2. We aimed to evaluate the association between social integration and face mask use during the COVID-19 pandemic in a random sample of households in Utsunomiya City, Greater Tokyo, Japan. Data included 645 adults in the Utsunomiya COVID-19 seROprevalence Neighborhood Association (U-CORONA) study, which was conducted after the first wave of the pandemic, between 14 June 2020 and 5 July 2020, in Utsunomiya City. Social integration before the pandemic was assessed by counting the number of social roles, based on the Cohen’s social network index. Face mask use before and during the pandemic was assessed by questionnaire, and participants were categorized into consistent mask users, new users, and current non-users. Multinomial logistic regression analysis was used to examine the association between lower social integration score and face mask use. To account for possible differential non-response bias, non-response weights were used. Of the 645 participants, 172 (26.7%) were consistent mask users and 460 (71.3%) were new users, while 13 (2.0%) were current non-users. Lower social integration level was positively associated with non-users (RRR: 1.76, 95% CI: 1.10, 2.82). Social integration may be important to promote face mask use.

## 1. Introduction

Patients infected with SARS-CoV-2 can transmit the virus even when they are still in pre-symptomatic phase [1,2,3]. A prior study reported that pre-symptomatic transmission accounted for ≈50% of all SARS-CoV-2 transmissions [1,2,3]. Thus, the Centers for Disease Control and Prevention in the US recommends universal face mask use as one of the important public health measures to control the spread of SARS-CoV-2 in the community [1]. A systematic review and meta-analysis reported the benefit of face mask use, which was attributed primarily to the effect of source control by reducing secondary infection from infected people, in reducing the risk of SARS-CoV-2 transmission [4]. In spite of the reported benefit, the prevalence of face mask use in public places is still not at a universal level. A recent survey showed that the prevalence of face mask use was ≈80% in the US [5,6] and ≈86% in Japan, although both the governments recommend face mask use in public [7].

Prior studies in the US have reported that male, younger age, race/ethnicity (i.e., being white compared to being black or Hispanic), political party (i.e., being a Republican), and living in rural areas were risk factors for not wearing a face mask [6,8,9]. A qualitative research in the US also found that barriers included discomfort associated with the use, misinformation, perceived low susceptibility, and perceptions of autonomy [10]. Interestingly, this study [10] and another one that was also conducted in the US [11] found that perceptions and the influence of people around them were one of the major determinants related to mask use. For example, the qualitative study reported that the major motivation for the use of face covering was protect or respect for other people [10]. Similarly, the other study reported that the perceived importance of others who wanted the study participants to wear a face covering was one of the major determinants of intention to wear a face covering [11]. Since the use of masks has different meanings in each culture, it is important to identify the characteristics of non-mask users in each culture in order to promote the use of masks, but studies examining the characteristics of mask non-users in Japan are lacking [12].

Social integration is defined as the degree to which an individual participates in various social relationships [13]. Following Durkheim’s pioneering study that examined the association between social integration and the prevalence of suicide, the social integration has been widely studied for its association with various health behaviors [13,14,15,16,17]. For example, Cohen et al. reported that higher social integration was associated with reductions in smoking and drinking [18]. Mechanisms by which social integration influences the adoption of favorable health behaviors include (1) increased sense of responsibility to others, (2) provision of multiple sources of information to influence health behaviors, and (3) provision of normative guidance from surrounding social networks [14,15,16]. However, the association between social integration and face mask use has been understudied.

Given the reported association between race/ethnicity and mask use, Japan’s relative racial and ethnic homogeneity, together with the high prevalence of mask use, make it an ideal setting to clarify the characteristics of people by their mask use behavior and to test the hypothesis that social integration is associated with face mask use. Furthermore, a prior study [19] has shown that vaccine refusers form localized clusters that interact less with others in the population, and they may be difficult to approach. Since mask non-users may form similar small clusters, it is necessary to use data from a random sample of population-based studies to identify small clusters of non-users and examine their characteristics. Therefore, we have set up our research in Japan to (1) clarify the characteristics of people by their mask use behavior (non-mask hygiene behaviors, lifestyle behaviors, past medical history, occupational characteristics, and health characteristics as determined by blood sampling) and to (2) evaluate the association between social integration and face mask use in a random sample of households in Utsunomiya City in Tochigi Prefecture, Greater Tokyo, Japan.

## 2. Methods

### 2.1. Sample

Data originated from the Utsunomiya COVID-19 seROprevalence Neighborhood Association (U-CORONA) study, which was conducted between 14 June 2020 and 5 July 2020 (after the first wave of the pandemic) in Utsunomiya City, Japan (Nawa et al., 2020). Invitations with a questionnaire attached were sent to 2290 people (1973 adults aged 18 years or older; and 317 children aged below 18 years) in 1000 households randomly selected from Utsunomiya City’s basic resident registry. A total of 649 adults and 104 children provided a valid response with written informed consent (response rate: 32.9%). Only data of these 649 adult participants were included, and samples without data on mask use were excluded (n = 4), which resulted in an analytical sample of 645 participants. Biomarkers were also measured for the 623 participants who were able to be present for blood tests.

### 2.2. Measures

#### 2.2.1. Independent Variables

##### Social Integration

Social integration before the pandemic was assessed by counting the number of social roles if the respondent regularly interacted with at least one person within that role before or during the pandemic, respectively. Based on the Cohen’s Social Network Index, the following types of social roles were assessed: spouse, child, parent, relatives, neighbor, colleague, group member (e.g., clubs, gyms, lessons, religious organizations), school friend, and others. A higher score indicates a higher social integration [20].

Social Capital

Individual-level social capital was measured using the following questions [21,22]: “Do you agree or disagree with the following statements? (1) People in your community can be trusted (social trust); (2) This community is close-knit (social ties); (3) People in your community are willing to help their neighbors (mutual aid) [21,22].” The responses were provided on a five-point Likert scale: 1 = strongly agree, 2 = somewhat agree, 3 = neither agree nor disagree, 4 = somewhat disagree, and 5 = strongly disagree. The inversed total score of the responses to these three questions was calculated and used as a social capital total score, with a higher score indicating higher social capital.

##### Covariates

Covariates included age, sex, education, household income, employment status, residential district, psychological distress, smoking, past medical history of asthma or other respiratory diseases, and social network size. These variables were used as categorical variables, except for age and social network size, which were used as continuous variables (Table 1). Information on age, sex, education, household income, employment status, psychological distress, smoking, and past medical history of asthma or other respiratory diseases was obtained by the questionnaire, while information on residential district was based on the basic resident registry.

Psychological distress was assessed by K6, a six-item scale used to screen for psychological distress, scoring from 0 to 24 with higher scores indicating more frequent psychological distress [23,24]. The Japanese version of the K6 was used in this study [23,24].

Social network size before the pandemic was measured using the question: “How many people do you usually meet or talk to?” The self-reported number of people for each item was used as the social network size before the pandemic.

##### Biomarkers

Blood samples were taken at the study site, and the levels of aspartate aminotransferase (AST), alanine aminotransferase (ALT), low-density lipoprotein cholesterol (LDL-C), high-density lipoprotein cholesterol (HDL-C), and high-sensitivity C-reactive protein (CRP) were measured by a commercial laboratory (SRL, Inc., Tokyo, Japan). The detection limit of CRP measurement was 0.05 mg/L. If the value was lower than 0.05 mg/L, the result “less than 0.05 mg/L” was returned, resulting in the value being unavailable. Therefore, the result of less than 0.05 mg/L was treated as 0.05 mg/L. Neutrophil and lymphocyte counts were measured using the automatic hematology analyzer Sysmex XN-1000 (Sysmex Corporation, Kobe, Japan). The neutrophil-to-lymphocyte ratio (NLR) was calculated by dividing neutrophil counts by lymphocyte counts, and log-transformed values were used to approximate a normal distribution.

#### 2.2.2. Dependent Variables

##### Mask Use

Mask use before and during the pandemic was assessed by questionnaire. Then, participants were categorized into three groups: consistent mask users (i.e., people who used masks both before and during the pandemic), new users (i.e., those who did not use masks before the pandemic but used them during the pandemic), current non-users (i.e., those who did not use masks during the pandemic, including those who did not use them both before and during the pandemic and those who used them before the pandemic but not during the pandemic).

### 2.3. Analysis

Multinomial logistic regression analysis was used to examine the relative risk ratio of being assigned to certain categories of mask use by a one-unit change in social integration score. The model was adjusted for age, sex, education, household income, employment status, residential district, psychological distress, smoking, past medical history of asthma or other respiratory diseases, and social network size before the pandemic. Furthermore, we also assessed an association between social capital and mask use by using multinomial logistic regression analysis. To account for possible differential non-response bias, we first estimated individual response probabilities using logistic regression with baseline data from the registry, including age, sex, distance to the clinic where the survey was conducted, residential district, and number of cohabitants of participants and non-participants. Then, non-response weights were calculated as the inverse of the predicted response probabilities and applied to multinomial logistic regression models.

To handle missing values on covariates, multiple imputation was used. Fifty imputed datasets were created. The results of the analyses using all the imputed datasets were combined using Rubin’s rules for multiple imputation (Rubin, 1987; Sterne et al., 2009). All analyses were conducted using STATA 14 (StataCorp LP, College Station, TX, USA).

## 3. Result

### 3.1. Demographic Characteristics of Study Participants by Mask Use Behavior

Of the 645 participants in the random sample of households in Utsunomiya City, we have identified 13 (2.0%) current non-users, including 11 who did not use them both before and during the pandemic and two who used them before the pandemic but not during. A total of 172 (26.7%) were consistent mask users and 460 (71.3%) were new users.

Table 1 displays the demographic characteristics of study participants by mask use behavior. About 36% of consistent mask users were male, while ≈50% of new users and ≈85% of current non-users were male. About 35% of consistent mask users had a psychological distress level of 5 or higher on the K6 scale, while ≈24% of new users and 23% of non-users had a psychological distress level of 5 or higher. The social integration level of current non-users was lower than that of consistent mask users and new users.

### 3.2. The Other Characteristics of People by Mask Use Behavior

Table 2 shows the average frequency of use of hygiene measures per day in a typical 2-week period before the pandemic by the type of mask use. Overall, hygiene measures such as hand washing, hand sanitizer use, and gargling were also more common in consistent mask users before the pandemic.

Table 3 displays the distribution of lifestyle behaviors before the pandemic by the type of mask use. About 11% of consistent mask users drank alcohol at least once a day, while ≈23% of new users and ≈31% of current non-users drank alcohol before the pandemic. Overall, consistent mask users slept less on both weekdays and weekends than new users and current non-users.

Table 4 shows the distribution of past medical history by the type of mask use. Overall, there was no difference in past medical history by type of mask use; however, the difference was observed in asthma or other respiratory diseases. About 13% of consistent mask users and ≈8% of new users had past medical history of asthma or other respiratory diseases, while ≈23% of current non-users had past medical history of asthma or other respiratory diseases.

Table 5 presents the distribution of work-related characteristics by the type of mask use. About 64% of consistent mask users were employed, while 58% of new users and ≈39% of current non-users were employed. Current non-users were characterized by a relatively high percentage of self-employed (≈23%) and not-working (≈39%).

In Table 6, we examined the difference in work-related characteristics by the type of mask uses among 433 participants who were employed or self-employed, after excluding those who were not working, were housemakers, and whose employment status was missing. The category with the largest percentage (accounting for 40–50% of both consistent mask users and new users) was the category with a workplace size of 5–99 people, while the category with 1–4 people was the most common category for non-users, accounting for about 40%.

Table 7 displays the health characteristics as determined by blood sampling by the type of mask use. Current non-users had higher AST values than consistent mask users and new users (AST in consistent mask users: 21.7; AST in new users: 22.8; AST in current non-users 50.3, *p* < 0.001). Similarly, current non-users had higher ALT values than consistent mask users and new users (ALT in consistent mask users: 19.4; ALT in new users: 22.3; ALT in current non-users 36.1, *p* = 0.008). There were no differences in LDL-C, HDL-C, CRP, or NLR by type of mask use.

### 3.3. Associations between Social Integration and Mask Use

Table 8 shows the weighted associations between social integration and mask use. Lower social integration was associated with a risk of being concurrent non-users (relative risk ratio (RRR): 1.73, 95% confidence interval (CI): 1.16, 2.57) before covariate adjustment. After covariate adjustment, lower social integration was still associated with the risk of being concurrent non-users (RRR: 1.76, 95% CI: 1.10, 2.82).

Table 9 presents the weighted associations between lower social capital and mask use. Lower social capital was associated with a risk of being concurrent non-users (RRR: 1.97, 95% CI: 0.85, 4.58) before covariate adjustment, although not reaching statistical significance. After covariate adjustment, lower social capital was marginally associated with a risk of being concurrent non-users (RRR: 2.60, 95% CI: 0.98, 6.90).

## 4. Discussion

In this study, we identified 13 (2.0%) current non-users, 172 (26.7%) consistent mask users, and 460 (71.3%) new users of masks. We also found that mask non-users were more likely to be male, self-employed, or not currently working with less psychological distress, less use of other hygiene measures, and more respiratory diseases (including asthma) but with a slightly altered liver function. Furthermore, we found that lower social integration was associated with the risk of being current non-users, after covariate adjustment.

Prior studies in the US have reported that male, younger age, being white, being a Republican in the US, and living in rural areas are risk factors for not wearing a mask [6,8,9]. Interestingly, other studies reported that major determinants of mask use and its proxy included perceptions and the influence of people around them, such as protection of or respect for other people [10], perceived importance of others who wanted the study participants to wear a face covering [11], and perceived norm in favor of mask use [25].

However, despite the social integration having been widely studied for its association with various health behaviors [13,14,15,16,18], the association between social integration and mask use has been not been sufficiently studied.

Previous studies have proposed several mechanisms by which social integration may affect the adoption of favorable health behaviors [14,15,16]. They include an increased sense of responsibility to others, provision of multiple sources of information to influence health behaviors, and provision of normative guidance from the surrounding social networks. In the case of mask wearing, since the benefits of masks come primary from reducing secondary infections from infected people, an increased sense of responsibility could be related to not transmitting the virus to others by wearing a mask. Furthermore, due to the relatively low prevalence of SARS-CoV-2 infection in Japan [26], people who are infected with the disease are often the focus of attention and are sometimes blamed [27,28]. This is more pronounced in areas with fewer infections. It has been reported that personal information, including details of the person’s behavior before and after infection and information on his/her workplaces, etc., was spread on the internet, leading to protest calls to workplaces [27,28]. Thus, some people with high social integration may be wearing masks so as not to bring about trouble to their friends and colleagues.

A social network study on polio vaccine hesitancy in India reported that households that refused vaccination had fewer outgoing social ties than households that accepted vaccination [19]. Using social network analysis, Christakis et al. reported that smoking and obesity may spread through social networks that surround people, and that the spread of people’s perceptions of social norms regarding the acceptability of smoking or obesity may contribute to the spread of smoking and obesity [29,30]. Thus, people with higher level of social integration may have similarly obtained multiple sources of information and normative guidance from the surrounding social networks [14,15,16], leading to mask-wearing behavior.

In this study, we found that the characteristics of mask non-users included male, self-employed, or not currently working with more respiratory diseases (including asthma) and slightly altered liver function. However, the detailed reasons for not using masks for non-mask users are unknown. We postulate that the reasons may include: (1) they had heard about the benefits of masks but did not trust the information, (2) they had heard about the benefits of masks but could not understand the information, (3) they received inaccurate (and often non-science-based) information that masks were not necessary and hence did not wear them accordingly, and (4) they did not know about the need for masks at all [31]. Since the measures to encourage non-mask users to wear masks vary depending on the reasons for not using masks, future research needs to clarify the reasons why they do not use masks.

In this study, we also found that lower social capital was marginally associated with the risk of being concurrent non-users. Coleman defined social capital as the resources available to the people in a community [32]. Our result may indicate that not only social integration but also social trust, which may be gained through social integration, is important [13].

This study has several limitations. First, the participation rate was low. Those who did not participate in this study might have lower social integration and use masks less frequently. This selection bias may have underestimated the association between low social integration and non-use of masks. To address this bias, non-response weights were calculated as the inverse of the predicted response probabilities and applied to multinomial logistic regression models. Second, the reason why non-mask users do not use masks may vary; thus, some participants may have specific reasons that are not related to social integration. Third, since the respondents were asked to self-report their current mask use, it is possible that those with high social integration were more likely to answer that they use masks because of social desirability bias. On the other hand, those with low social integration were less influenced by the social norm of using masks. Thus, they may have been less affected by social desirability bias and were less likely to answer that they use masks. This may have led to an overestimation of the relationship between low social integration and non-use of masks. Finally, because social integration before the SARS-CoV-2 pandemic was asked by recall, there may have been a measurement error in social integration, and the relationship between low social integration and non-use of masks may have been underestimated.

Nonetheless, our study has implications for public health. The government currently encourages the public to use face masks through information and messages from government websites, newspapers, and television news programs [1,7]. However, given our finding that people who do not use masks are less socially integrated, current approaches may not be effective in promoting mask use among non-mask users. It is important to change the behavior of non-mask users in order to achieve a universal level of mask use and contain the further spread of the infection [4]. Thus, social integration policy may be needed to promote mask use, which is an important measure to prevent the spread of SARS-CoV-2.

## 5. Conclusions

In conclusion, we found that lower social integration level was associated with the risk of being a current face mask non-user. Further research is needed to promote the use of masks, especially among those who do not use masks.

## Figures and Tables

**Table 1:** Demographic characteristics of study participants by mask use behavior (n = 645).

Variable		Consistent Mask Users (n = 172)	New Users (n = 460)	Current Non-Users (n = 13)	*p*-Value
		N (%) or Mean (SD)	N (%) or Mean (SD)	N (%) or Mean (SD)	
Age (in years)	Mean (SD)	50.9 (18.0)	52.1 (16.6)	54.4 (18.8)	0.649
Sex	Male	62 (36.1)	231 (50.2)	11 (84.6)	**<0.001**
	Female	110 (64.0)	229 (49.8)	2 (15.4)	
BMI	Mean (SD)	22.7 (3.1)	23.5 (3.9)	22.7 (3.7)	**0.047**
Residential district	Center	71 (41.3)	166 (36.1)	7 (53.9)	0.478
	East	69 (40.1)	188 (40.9)	4 (30.8)	
	South	32 (18.6)	106 (23.0)	2 (15.4)	
Number of cohabitants	1	26 (15.1)	51 (11.1)	2 (15.4)	0.185
	2	64 (37.2)	140 (30.4)	4 (30.8)	
	≥3	82 (47.7)	269 (58.5)	7 (53.9)	
Education	High school or less	73 (42.4)	173 (37.6)	7 (53.9)	0.245
	Some college	38 (22.1)	108 (23.5)	1 (7.7)	
	College or more	55 (32.0)	167 (36.3)	4 (30.8)	
	Others	3 (1.7)	1 (0.2)	0 (0.0)	
	Missing	3 (1.7)	11 (2.4)	1 (7.7)	
Annual household income (yen)	<3 million	42 (24.4)	91 (19.8)	5 (38.5)	0.417
	3–6 million	48 (27.9)	128 (27.8)	4 (30.8)	
	6–10 million	50 (29.1)	125 (27.2)	1 (7.7)	
	≥10 million	18 (10.5)	58 (12.6)	1 (7.7)	
	unknown/missing	14 (8.1)	58 (12.6)	2 (15.4)	
Psychological distress	Yes (K6: 5+ )	60 (34.9)	109 (23.7)	3 (23.1)	**<0.001**
	No (K6: <5)	111 (64.5)	341 (74.1)	8 (61.5)	
	Missing	1 (0.6)	10 (2.2)	2 (15.4)	
Social integration	Mean (SD)	4.1 (1.7)	4.0 (1.7)	2.6 (1.2)	**0.011**
Social network size	Mean (SD)	25.1 (44.5)	22.8 (39.5)	26.2 (32.4)	0.802
Social capital score	Mean (SD)	2.3 (0.8)	2.4 (0.8)	2.0 (1.2)	0.183

Bold indicates *p* < 0.05.

**Table 2:** Average frequency of use of hygiene measures per day in a typical 2-week period before the pandemic by mask use behavior (n = 645).

Variable		Consistent Mask Users (n = 172)	New Users (n = 460)	Current Non-Users (n = 13)	*p*-Value
		N (%)	N (%)	N (%)	
Washing hand (times per day)	0	3 (1.7)	15 (3.3)	1 (7.7)	**0.001**
	1–3	36 (20.9)	169 (36.7)	3 (23.1)	
	4–6	54 (31.4)	121 (26.3)	5 (38.5)	
	7–9	20 (11.6)	63 (13.7)	1 (7.7)	
	≥10	59 (34.3)	92 (20.0)	3 (23.1)	
	Missing	0 (0.0)	0 (0.0)	0 (0.0)	
Hand sanitizer (times per day)	0	66 (38.4)	344 (74.8)	8 (61.5)	**<0.001**
	1	14 (8.1)	42 (9.1)	2 (15.4)	
	2–3	34 (19.8)	43 (9.4)	1 (7.7)	
	≥4	58 (33.7)	29 (6.3)	2 (15.4)	
	Missing	0 (0.0)	2 (0.4)	0 (0.0)	
Gargling (times per day)	0	25 (14.5)	121 (26.3)	4 (30.8)	**0.011**
	1	36 (20.9)	120 (26.1)	2 (15.4)	
	2–3	77 (44.8)	149 (32.4)	5 (38.5)	
	≥4	34 (19.8)	70 (15.2)	2 (15.4)	
	Missing	0 (0.0)	0 (0.0)	0 (0.0)	

Bold indicates *p* < 0.05.

**Table 3:** Distribution of lifestyle behaviors before the pandemic by mask use behavior (n = 645).

Variable		Consistent Mask Users (n = 172)	New Users (n = 460)	Current Non-Users (n = 13)	*p*-Value
		N (%)	N (%)	N (%)	
Frequency of Drinking alcohol	Never	71 (41.3)	148 (32.2)	6 (46.2)	**0.036**
	little	25 (14.5)	57 (12.4)	1 (7.7)	
	≥1 per month	22 (12.8)	38 (8.3)	0 (0.0)	
	≥1 per week	33 (19.2)	105 (22.8)	2 (15.4)	
	≥1 per day	19 (11.1)	107 (23.3)	4 (30.8)	
	Missing	2 (1.2)	5 (1.1)	0 (0.0)	
Smoking tobacco	Yes	17 (9.9)	59 (12.8)	4 (30.8)	0.174
	No or quitted	153 (89.0)	390 (84.8)	9 (69.2)	
	Missing	2 (1.2)	11 (2.4)	0 (0.0)	
Physical activity per week (days)	0	75 (43.6)	204 (44.4)	6 (46.2)	0.132
	1–4	72 (41.9)	185 (40.2)	4 (30.8)	
	5–7	25 (14.5)	67 (14.6)	2 (15.4)	
	Missing	0 (0.0)	4 (0.9)	1 (7.7)	
Sleep duration on weekdays (hours)	<6	57 (33.1)	113 (24.6)	1 (7.7)	**0.001**
	6–8	111 (64.5)	320 (69.6)	9 (69.2)	
	8–10	3 (1.7)	24 (5.2)	2 (15.4)	
	≥10	1 (0.6)	0 (0.0)	0 (0.0)	
	Missing	0 (0.0)	3 (0.7)	1 (7.7)	
Sleep duration on weekend (hours)	<6	30 (17.4)	58 (12.6)	0 (0.0)	**0.013**
	6–8	115 (66.9)	337 (73.3)	8 (61.5)	
	8–10	23 (13.4)	58 (12.6)	4 (30.8)	
	≥10	3 (1.7)	5 (1.1)	0 (0.0)	
	Missing	1 (0.6)	2 (0.4)	1 (7.7)	

Bold indicates *p* < 0.05.

**Table 4:** Distribution of past medical history by mask use behavior (n = 645).

Variable		Consistent Mask Users (n = 172)	New Users (n = 460)	Current Non-Users (n = 13)	*p*-Value
		N (%)	N (%)	N (%)	
Allergic diseases	Yes	71 (41.3)	168 (36.5)	3 (23.1)	0.303
	No	101 (58.7)	292 (63.5)	10 (76.9)	
Asthma or other respiratory diseases	Yes	23 (13.4)	38 (8.3)	3 (23.1)	**0.044**
	No	149 (86.6)	422 (91.7)	10 (76.9)	
Cardiac diseases	Yes	7 (4.1)	18 (3.9)	1 (7.7)	0.791
	No	165 (95.9)	442 (96.1)	12 (92.3)	
Kidney diseases	Yes	4 (2.3)	7 (1.5)	1 (7.7)	0.233
	No	168 (97.7)	453 (98.5)	12 (92.3)	
Immune diseases	Yes	5 (2.9)	7 (1.5)	0 (0.0)	0.457
	No	167 (97.1)	453 (98.5)	13 (100.0)	
Diabetes mellitus	Yes	14 (8.1)	40 (8.7)	2 (15.4)	0.670
	No	158 (91.9)	420 (91.3)	11 (84.6)	
Malignant diseases	Yes	8 (4.7)	17 (3.7)	2 (15.4)	0.109
	No	164 (95.4)	443 (96.3)	11 (84.6)	
Arthritis	Yes	11 (6.4)	14 (3.0)	0 (0.0)	0.116
	No	161 (93.6)	446 (97.0)	13 (100.0)	
Gastrointestinal diseases	Yes	14 (8.1)	38 (8.3)	3 (23.1)	0.165
	No	158 (91.9)	422 (91.7)	10 (76.9)	
Mental diseases	Yes	6 (3.5)	19 (4.1)	0 (0.0)	0.714
	No	166 (96.5)	441 (95.9)	13 (100.0)	
Alcohol and drug abuse	Yes	1 (0.6)	0 (0.0)	0 (0.0)	0.252
	No	171 (99.4)	460 (100.0)	13 (100.0)	
Tuberculosis	Yes	2 (1.2)	6 (1.3)	1 (7.7)	0.146
	No	170 (98.8)	454 (98.7)	12 (92.3)	

Bold indicates *p* < 0.05.

**Table 5:** Distribution of work-related characteristics by mask use behavior (n = 645).

Variable		Consistent Mask Users (n = 172)	New Users (n = 460)	Current Non-Users (n = 13)	*p*-Value
		N (%)	N (%)	N (%)	
Employment	Employed	110 (64.0)	266 (57.8)	5 (38.5)	**0.039**
	Self-employed	13 (7.6)	36 (7.8)	3 (23.1)	
	Not working	21 (12.2)	72 (15.7)	5 (38.5)	
	Housemakers	28 (16.3)	79 (17.2)	0 (0.0)	
	Missing	0 (0.0)	7 (1.5)	0 (0.0)	

Bold indicates *p* < 0.05.

**Table 6:** Distribution of workplace size and job types by mask use behavior among workers (n = 433) ^a^.

Variable		Consistent Mask Users (n = 123)	New Users (n = 302)	Current Non-Users (n = 8)	*p*-Value
		N (%)	N (%)	N (%)	
Workplace Size (number of people in the workplace)	1–4	18 (14.6)	39 (12.9)	3 (37.5)	**0.034**
	5–99	62 (50.4)	122 (40.4)	1 (12.5)	
	100–499	14 (11.4)	51 (16.9)	2 (25.0)	
	≥500	26 (21.1)	60 (19.9)	1 (12.5)	
	Government offices (including local government)	1 (0.8)	20 (6.6)	0 (0.0)	
	Missing	2 (1.6)	10 (3.3)	1 (12.5)	
Job types	Management	11 (8.9)	37 (12.3)	0 (0.0)	0.220
	Professional work	29 (23.6)	60 (19.9)	0 (0.0)	
	Office work	19 (15.5)	43 (14.2)	0 (0.0)	
	Service and sales	18 (14.6)	38 (12.6)	1 (12.5)	
	Others	27 (22.0)	80 (26.5)	3 (37.5)	
	Missing	19 (15.5)	44 (14.6)	4 (50.0)	

^a^ Those who were not working, were housemakers, and those whose employment status was missing were excluded. Bold indicates *p* < 0.05.

**Table 7:** The health characteristics as determined by blood sampling by mask use behavior (n = 623) ^a^.

Variable	Consistent Mask Users (n = 166)	New Users (n = 446)	Current Non-Users (n = 11)	*p*-Value
	N (%)	N (%)	N (%)	
AST	21.7 (9.5)	22.8 (11.8)	50.3 (62.3)	**<0.001**
ALT	19.4 (11.8)	22.3 (20.2)	36.1 (27.0)	**0.008**
LDL-C	116.9 (30.9)	113.9 (27.4)	108.5 (27.7)	0.399
HDL-C	62.1 (14.4)	60.9 (16.1)	59.7 (12.1)	0.661
NLR	0.54 (0.41)	0.52 (0.41)	0.34 (0.35)	0.414
CRP	1.07 (3.33)	0.87 (1.96)	1.14 (1.29)	0.627

^a^ Participants with missing values in blood test results (n = 22) were excluded. AST, aspartate aminotransferase; ALT, alanine aminotransferase; LDL-C, low-density lipoprotein cholesterol; HDL-C, high-density lipoprotein cholesterol; NLR, neutrophil-to-lymphocyte ratio; CRP, C-reactive protein. NLR was log-transformed to approximate a normal distribution. Bold indicates *p* < 0.05.

**Table 8:** Weighted associations between lower social integration and mask use behavior in the imputed dataset (n = 645).

	Consistent Mask Users (n = 172)	New Users (n = 460)	Current Non-Users (n = 13)
	RRR (95% CI)	Reference	RRR (95% CI)
Crude model ^a^			
Low social integration	0.97 (0.88, 1.08)	1.00 (Reference)	**1.73** **(1.16, 2.57)**
Adjusted model ^a,b^			
Low social integration	1.00 (0.90, 1.12)	1.00 (Reference)	**1.76** **(1.10, 2.82)**

^a^ Social integration score was reversed so that the effect of a one-unit increase can be interpreted as the effect of a one-point decrease. ^b^ Adjusted for age, sex, education, household income, employment status, residential district, psychological distress, smoking, past medical history of asthma or other respiratory diseases and social network size, using a multinomial logistic regression. Bold indicates *p* < 0.05. RRR, relative risk ratio.

**Table 9:** Weighted associations between lower social capital and mask use behavior in imputed dataset (n = 645).

Heading	Consistent Mask Users (n = 172)	New Users (n = 460)	Current Non-Users (n = 13)
	RRR (95% CI)	Reference	RRR (95% CI)
Crude model			
Low social capital score ^a^	1.08 (0.86, 1.36)	1.00 (Reference)	1.97 (0.85, 4.58)
Adjusted model ^b^			
Low social capital score ^a^	1.02 (0.79, 1.32)	1.00 (Reference)	2.60 (0.98, 6.90)

^a^ Social capital score was reversed and scaled so that the effect of a one-unit increase can be interpreted as the effect of a one interquartile range (IQR) decrease. ^b^ Adjusted for age, sex, education, household income, employment status, residential district, psychological distress, smoking, past medical history of asthma or other respiratory diseases, using a multinomial logistic regression. Bold indicates *p* < 0.05. RRR, relative risk ratio.

## Data Availability

The data presented in this study are available on request from the corresponding author.

## References

[1] Honein M.A., Christie A., Rose D.A., Brooks J.T., Meaney-Delman D., Cohn A., Sauber-Schatz E.K., Walker A., McDonald L.C., Liburd L.C. (2020). Summary of Guidance for Public Health Strategies to Address High Levels of Community Transmission of SARS-CoV-2 and Related Deaths, December 2020. MMWR Morb Mortal Wkly Rep..

[2] Moghadas S.M., Fitzpatrick M.C., Sah P., Pandey A., Shoukat A., Singer B.H., Galvani A.P. (2020). The implications of silent transmission for the control of COVID-19 outbreaks. Proc. Natl. Acad. Sci. USA.

[3] He X., Lau E.H.Y., Wu P., Deng X., Wang J., Hao X., Lau Y.C., Wong J.Y., Guan Y., Tan X. (2020). Temporal dynamics in viral shedding and transmissibility of COVID-19. Nat. Med..

[4] Chu D.K., Akl E.A., Duda S., Solo K., Yaacoub S., Schunemann H.J. (2020). Physical distancing, face masks, and eye protection to prevent person-to-person transmission of SARS-CoV-2 and COVID-19: A systematic review and meta-analysis. Lancet.

[5] YouGov COVID-19 Behaviour Changes Tracker: Wearing a Face Mask When in Public Places. https://yougov.co.uk/topics/international/articles-reports/2020/03/17/personal-measures-taken-avoid-covid-19.

[6] A Detailed Map of Who Is Wearing Masks in the U.S. The New York Times.

[7] Ministry of Health, Labour and Welfare, Japan Winter Corona Measures. https://www.mhlw.go.jp/stf/newpage_14992.html.

[8] Haischer M.H., Beilfuss R., Hart M.R., Opielinski L., Wrucke D., Zirgaitis G., Uhrich T.D., Hunter S.K. (2020). Who is wearing a mask? Gender-, age-, and location-related differences during the COVID-19 pandemic. PLoS ONE.

[9] Pew Research Center Republicans, Democrats Move Even Further Apart in Coronavirus Concerns. https://www.pewresearch.org/politics/2020/06/25/republicans-democrats-move-even-further-apart-in-coronavirus-concerns/.

[10] Shelus V.S., Frank S.C., Lazard A.J., Higgins I.C.A., Pulido M., Richter A.P.C., Vandegrift S.M., Vereen R.N., Ribisl K.M., Hall M.G. (2020). Motivations and Barriers for the Use of Face Coverings during the COVID-19 Pandemic: Messaging Insights from Focus Groups. Int. J. Environ. Res. Public Health.

[11] Barile J.P., Guerin R.J., Fisher K.A., Tian L.H., Okun A.H., Vanden Esschert K.L., Jeffers A., Gurbaxani B.M., Thompson W.W., Prue C.E. (2021). Theory-based Behavioral Predictors of Self-reported Use of Face Coverings in Public Settings during the COVID-19 Pandemic in the United States. Ann. Behav. Med..

[12] Van der Westhuizen H.M., Kotze K., Tonkin-Crine S., Gobat N., Greenhalgh T. (2020). Face coverings for covid-19: From medical intervention to social practice. BMJ.

[13] Cohen S., Underwood L.G., Gottlieb B.H. (2000). Social Support Measurement and Intervention: A Guide for Health and Social Scientists.

[14] Berkman L.F., Glass T., Brissette I., Seeman T.E. (2000). From social integration to health: Durkheim in the new millennium. Soc. Sci. Med..

[15] Cohen S. (2004). Social relationships and health. Am. Psychol..

[16] Berkman L.F., Kawachi I., Glymour M.M. (2014). Social Epidemiology.

[17] Durkheim E. (1951). Suicide: A Study in Sociology.

[18] Cohen S., Lemay E.P. (2007). Why would social networks be linked to affect and health practices?. Health Psychol..

[19] Onnela J.P., Landon B.E., Kahn A.L., Ahmed D., Verma H., O’Malley A.J., Bahl S., Sutter R.W., Christakis N.A. (2016). Polio vaccine hesitancy in the networks and neighborhoods of Malegaon, India. Soc. Sci. Med..

[20] Cohen S., Doyle W.J., Skoner D.P., Rabin B.S., Gwaltney J.M. (1997). Social ties and susceptibility to the common cold. JAMA.

[21] Jung M., Lin L., Viswanath K. (2013). Associations between health communication behaviors, neighborhood social capital, vaccine knowledge, and parents’ H1N1 vaccination of their children. Vaccine.

[22] Sampson R.J., Raudenbush S.W., Earls F. (1997). Neighborhoods and violent crime: A multilevel study of collective efficacy. Science.

[23] Furukawa T.A., Kawakami N., Saitoh M., Ono Y., Nakane Y., Nakamura Y., Tachimori H., Iwata N., Uda H., Nakane H. (2008). The performance of the Japanese version of the K6 and K10 in the World Mental Health Survey Japan. Int. J. Methods Psychiatr. Res..

[24] Kessler R.C., Andrews G., Colpe L.J., Hiripi E., Mroczek D.K., Normand S.L., Walters E.E., Zaslavsky A.M. (2002). Short screening scales to monitor population prevalences and trends in non-specific psychological distress. Psychol. Med..

[25] Fisher K.A., Barile J.P., Guerin R.J., Vanden Esschert K.L., Jeffers A., Tian L.H., Garcia-Williams A., Gurbaxani B., Thompson W.W., Prue C.E. (2020). Factors Associated with Cloth Face Covering Use Among Adults During the COVID-19 Pandemic-United States, April and May 2020. MMWR Morb. Mortal Wkly Rep..

[26] World Health Organization (WHO) WHO Coronavirus Disease (COVID-19) Dashboard. https://covid19.who.int/.

[27] The Asahi Shimbun Company Slander Continues for First Infected Person in Iwate. https://www.asahi.com/articles/ASN813J27N70ULUC00B.html.

[28] The Asahi Shimbun Company Yamanashi Prefecture to Take Measures Against Slander of Infected Women. https://www.asahi.com/articles/ASN576V6GN57UZOB00Y.html.

[29] Christakis N.A., Fowler J.H. (2007). The spread of obesity in a large social network over 32 years. N. Engl. J. Med..

[30] Christakis N.A., Fowler J.H. (2008). The collective dynamics of smoking in a large social network. N. Engl. J. Med..

[31] McGuire W.J., Rice R.E., Atkin C.K. (2013). McGuire’s Classic Input-Output Framework for Constructing Persuasive Messages. Public Communication Campaigns.

[32] Coleman J.S. (1990). Foundations of Social Theory.

